# *catena*-Poly[[di-μ-iodido-dicopper(I)(*Cu*—*Cu*)]bis­(μ-4,4′-di-3-pyridyl-2,2′-disulfanediyldipyrimidine)]

**DOI:** 10.1107/S1600536809052325

**Published:** 2009-12-12

**Authors:** Hai-Bin Zhu

**Affiliations:** a School of Chemistry and Chemical Engineering, Southeast University, Nanjing 211189, People’s Republic of China, and School of Material Science and Engineering, Southeast University, Nanjing 211189, People’s Republic of China

## Abstract

The title complex, [Cu_2_I_2_(C_18_H_12_N_6_S_2_)_2_]_*n*_, contains a Cu_2_I_2_ core with a Cu—Cu distance of 2.6935 (14) Å. The Cu^I^ atom is coordinated by two bridging 4,4′-di-3-pyridyl-2,2′-disulfanediyldipyrimidine ligands and two bridging I atoms, forming a double chain.

## Related literature

For coordination polymers with 4,4′-dipyridine­disulfide, see: Horikoshi & Mochida (2006 ▶). For coordination polymers with 2,2′-dithio­bis(4-pyridin-4-yl-pyrimidine), see: Zhu *et al.* (2009 ▶). For the structure of free 2,2′-dithio­bis(3-pyridin-4-yl-pyrimidine), see: Ji *et al.* (2009 ▶).
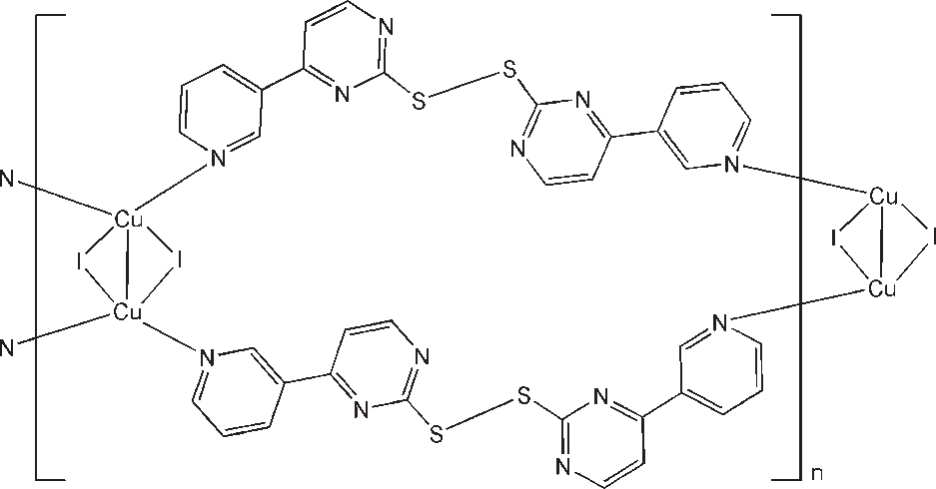


## Experimental

### 

#### Crystal data

[Cu_2_I_2_(C_18_H_12_N_6_S_2_)_2_]
*M* *_r_* = 1133.86
Triclinic, 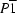

*a* = 8.5561 (6) Å
*b* = 10.7702 (8) Å
*c* = 11.9045 (8) Å
α = 98.110 (1)°
β = 107.193 (1)°
γ = 96.449 (1)°
*V* = 1023.66 (13) Å^3^
*Z* = 1
Mo *K*α radiation
μ = 2.80 mm^−1^
*T* = 298 K
0.19 × 0.15 × 0.12 mm

#### Data collection

Bruker APEXII CCD diffractometer
Absorption correction: multi-scan (*SADABS*; Bruker, 2001 ▶) *T*_min_ = 0.611, *T*_max_ = 0.715
5395 measured reflections
3568 independent reflections
2956 reflections with *I* > 2σ(*I*)
*R*_int_ = 0.097

#### Refinement

*R*[*F* ^2^ > 2σ(*F* ^2^)] = 0.041
*wR*(*F* ^2^) = 0.108
*S* = 0.99
3568 reflections
253 parameters
H-atom parameters constrained
Δρ_max_ = 1.28 e Å^−3^
Δρ_min_ = −1.03 e Å^−3^

### 

Data collection: *APEX2* (Bruker, 2007 ▶); cell refinement: *SAINT-Plus* (Bruker, 2007 ▶); data reduction: *SAINT-Plus*; program(s) used to solve structure: *SHELXS97* (Sheldrick, 2008 ▶); program(s) used to refine structure: *SHELXL97* (Sheldrick, 2008 ▶); molecular graphics: *SHELXTL* (Sheldrick, 2008 ▶) and *DIAMOND* (Brandenburg, 1999 ▶); software used to prepare material for publication: *SHELXTL*.

## Supplementary Material

Crystal structure: contains datablocks I, global. DOI: 10.1107/S1600536809052325/hy2260sup1.cif

Structure factors: contains datablocks I. DOI: 10.1107/S1600536809052325/hy2260Isup2.hkl

Additional supplementary materials:  crystallographic information; 3D view; checkCIF report

## Figures and Tables

**Table 1:** Selected bond lengths (Å)

Cu1—I1	2.6550 (7)
Cu1—I1^i^	2.6579 (8)
Cu1—N1	2.037 (4)
Cu1—N6^ii^	2.060 (4)

Symmetry codes: (i) 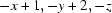; (ii) 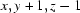.

**Fig. 1.**
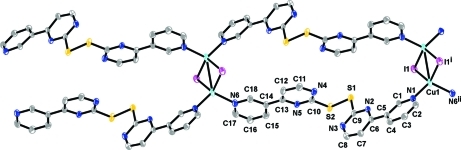
The structure of the title compound with 30% probability displacement ellipsoids. H atoms have been omitted for clarity. [Symmetry codes: (i) -x + 1, -y + 2, -z; (ii) x, y + 1, z - 1.]

**Fig. 2.**
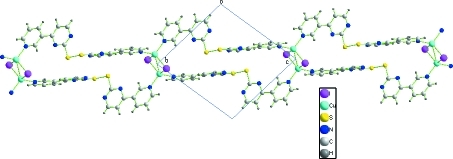
The one-dimensional double chain viewed along the a axis.

## supplementary crystallographic information

### Comment

In recent years, heterocyclic disulfide ligands have received increasing attention because of their conformationally defined dihedral angle (Horikoshi & Mochida, 2006). As continuation of our previous research (Zhu *et al.*, 2009), we report here a copper(I) coordination polymer with a 2,2'-dithiobis(3-pyridin-4-ylpyrimidine) (*L*) ligand.

The Cu^I^ atom in the title complex has a tetrahedral coordination geometry completed by two N atoms from two different *L* ligands and two bridging I atoms (Fig. 1 and Table 1). The C—S—S—C torsion angle of 81.2 (2)° in *L* is almost identical with the free molecule (Ji *et al.*, 2009). Alternative linkings of one Cu_2_I_2_ core and two bridging *L* ligands generate a one-dimensional double chain (Fig. 2).

### Experimental

A CH_2_Cl_2_ solution (5 ml) of ligand *L* (0.1 mmol) was slowly added into a CuI (0.1 mmol) solution in acetonitrile (10 ml). The mixture was kept on standing for 3 d to give single crystals suitable for X-ray diffraction analysis.

### Refinement

H atoms were positioned geometrically and refined as riding atoms, with C—H = 0.93 Å and *U*_iso_(H) = 1.2*U*_eq_(C). The highest residual electron density was found 0.98 Å from I1 and the deepest hole 0.92 Å from I1.

### Crystal data

**Table:**

[Cu_2_I_2_(C_18_H_12_N_6_S_2_)_2_]	*Z* = 1
*M**_r_* = 1133.86	*F*(000) = 552
Triclinic, *P*1	*D*_x_ = 1.839 Mg m^−^^3^
Hall symbol: -P 1	Mo *K*α radiation, λ = 0.71073 Å
*a* = 8.5561 (6) Å	Cell parameters from 3568 reflections
*b* = 10.7702 (8) Å	θ = 2.3–25.5°
*c* = 11.9045 (8) Å	µ = 2.80 mm^−^^1^
α = 98.110 (1)°	*T* = 298 K
β = 107.193 (1)°	Block, yellow
γ = 96.449 (1)°	0.19 × 0.15 × 0.12 mm
*V* = 1023.66 (13) Å^3^	

### Data collection

**Table:**

Bruker APEXII CCD diffractometer	3568 independent reflections
Radiation source: fine-focus sealed tube	2956 reflections with *I* > 2σ(*I*)
graphite	*R*_int_ = 0.097
φ and ω scans	θ_max_ = 25.0°, θ_min_ = 1.8°
Absorption correction: multi-scan (*SADABS*; Bruker, 2001)	*h* = −7→10
*T*_min_ = 0.611, *T*_max_ = 0.715	*k* = −12→12
5395 measured reflections	*l* = −14→13

### Refinement

**Table:**

Refinement on *F*^2^	Primary atom site location: structure-invariant direct methods
Least-squares matrix: full	Secondary atom site location: difference Fourier map
*R*[*F*^2^ > 2σ(*F*^2^)] = 0.041	Hydrogen site location: inferred from neighbouring sites
*wR*(*F*^2^) = 0.108	H-atom parameters constrained
*S* = 0.99	*w* = 1/[σ^2^(*F*_o_^2^) + (0.0541*P*)^2^] where *P* = (*F*_o_^2^ + 2*F*_c_^2^)/3
3568 reflections	(Δ/σ)_max_ = 0.001
253 parameters	Δρ_max_ = 1.28 e Å^−^^3^
0 restraints	Δρ_min_ = −1.03 e Å^−^^3^

### Fractional atomic coordinates and isotropic or equivalent isotropic displacement parameters (Å^2^)

**Table:**

	*x*	*y*	*z*	*U*_iso_*/*U*_eq_	
I1	0.31696 (4)	0.97725 (3)	0.10525 (3)	0.04697 (15)	
Cu1	0.38873 (9)	0.92391 (6)	−0.09670 (6)	0.0503 (2)	
S1	0.13540 (17)	0.55124 (12)	0.27577 (10)	0.0447 (3)	
S2	0.02067 (16)	0.43336 (14)	0.35509 (12)	0.0498 (3)	
N2	0.2094 (5)	0.5182 (3)	0.0809 (3)	0.0340 (8)	
C5	0.3021 (5)	0.5258 (4)	−0.0900 (4)	0.0328 (10)	
N3	0.0948 (5)	0.3267 (4)	0.1271 (4)	0.0426 (10)	
N1	0.3815 (5)	0.7322 (4)	−0.1296 (3)	0.0387 (9)	
C9	0.1455 (5)	0.4513 (4)	0.1466 (4)	0.0340 (10)	
N5	0.1235 (5)	0.2820 (4)	0.4999 (3)	0.0381 (9)	
C1	0.3142 (6)	0.6574 (4)	−0.0705 (4)	0.0356 (10)	
H1A	0.2728	0.6955	−0.0127	0.043*	
C6	0.2284 (5)	0.4523 (4)	−0.0183 (4)	0.0330 (10)	
C10	0.1797 (6)	0.3566 (5)	0.4357 (4)	0.0392 (11)	
C3	0.4346 (6)	0.5483 (5)	−0.2380 (4)	0.0436 (11)	
H3B	0.4772	0.5130	−0.2963	0.052*	
C13	0.2302 (6)	0.2187 (4)	0.5638 (4)	0.0381 (10)	
C12	0.3936 (6)	0.2331 (5)	0.5625 (4)	0.0480 (13)	
H12A	0.4704	0.1894	0.6067	0.058*	
C4	0.3639 (6)	0.4719 (5)	−0.1776 (4)	0.0411 (11)	
H4A	0.3572	0.3842	−0.1951	0.049*	
N4	0.3312 (5)	0.3777 (4)	0.4277 (3)	0.0453 (10)	
C11	0.4371 (6)	0.3147 (5)	0.4933 (5)	0.0515 (13)	
H11A	0.5461	0.3261	0.4925	0.062*	
C2	0.4419 (6)	0.6772 (5)	−0.2117 (4)	0.0418 (11)	
H2B	0.4909	0.7284	−0.2528	0.050*	
C8	0.1115 (6)	0.2636 (5)	0.0281 (5)	0.0457 (12)	
H8A	0.0766	0.1761	0.0087	0.055*	
C7	0.1775 (6)	0.3213 (4)	−0.0466 (4)	0.0416 (11)	
H7A	0.1883	0.2739	−0.1148	0.050*	
C14	0.1671 (6)	0.1341 (4)	0.6336 (4)	0.0380 (10)	
C15	−0.0011 (6)	0.1112 (5)	0.6209 (4)	0.0475 (12)	
H15A	−0.0761	0.1500	0.5691	0.057*	
C16	−0.0545 (7)	0.0301 (5)	0.6864 (5)	0.0532 (14)	
H16A	−0.1663	0.0141	0.6796	0.064*	
C18	0.2701 (7)	0.0733 (5)	0.7125 (4)	0.0456 (12)	
H18A	0.3827	0.0880	0.7217	0.055*	
C17	0.0554 (7)	−0.0264 (5)	0.7604 (4)	0.0503 (13)	
H17A	0.0161	−0.0825	0.8023	0.060*	
N6	0.2191 (5)	−0.0053 (4)	0.7765 (3)	0.0442 (10)	

### Atomic displacement parameters (Å^2^)

**Table:**

	*U*^11^	*U*^22^	*U*^33^	*U*^12^	*U*^13^	*U*^23^
I1	0.0504 (2)	0.0489 (2)	0.0526 (2)	0.00536 (15)	0.02987 (17)	0.01773 (16)
Cu1	0.0647 (4)	0.0437 (4)	0.0576 (4)	0.0113 (3)	0.0319 (3)	0.0285 (3)
S1	0.0595 (8)	0.0464 (7)	0.0346 (6)	0.0117 (6)	0.0179 (6)	0.0187 (5)
S2	0.0478 (7)	0.0725 (9)	0.0424 (7)	0.0181 (7)	0.0204 (6)	0.0338 (7)
N2	0.039 (2)	0.034 (2)	0.0308 (19)	0.0039 (16)	0.0112 (16)	0.0143 (16)
C5	0.031 (2)	0.036 (2)	0.031 (2)	0.0029 (19)	0.0078 (18)	0.0140 (19)
N3	0.045 (2)	0.040 (2)	0.049 (2)	0.0049 (18)	0.0167 (19)	0.0225 (19)
N1	0.044 (2)	0.038 (2)	0.038 (2)	0.0031 (17)	0.0182 (18)	0.0159 (17)
C9	0.035 (2)	0.040 (3)	0.028 (2)	0.008 (2)	0.0081 (19)	0.0147 (19)
N5	0.042 (2)	0.046 (2)	0.0293 (19)	0.0054 (18)	0.0114 (17)	0.0156 (17)
C1	0.042 (3)	0.035 (2)	0.036 (2)	0.007 (2)	0.018 (2)	0.012 (2)
C6	0.033 (2)	0.034 (2)	0.033 (2)	0.0050 (19)	0.0091 (19)	0.0131 (19)
C10	0.044 (3)	0.045 (3)	0.030 (2)	0.004 (2)	0.012 (2)	0.012 (2)
C3	0.046 (3)	0.053 (3)	0.036 (2)	0.008 (2)	0.018 (2)	0.008 (2)
C13	0.046 (3)	0.040 (3)	0.029 (2)	0.004 (2)	0.011 (2)	0.012 (2)
C12	0.041 (3)	0.060 (3)	0.047 (3)	0.012 (2)	0.010 (2)	0.030 (3)
C4	0.046 (3)	0.039 (3)	0.039 (3)	0.005 (2)	0.015 (2)	0.008 (2)
N4	0.043 (2)	0.057 (3)	0.039 (2)	0.006 (2)	0.0127 (18)	0.021 (2)
C11	0.038 (3)	0.069 (4)	0.051 (3)	0.001 (3)	0.013 (2)	0.027 (3)
C2	0.046 (3)	0.048 (3)	0.036 (3)	0.000 (2)	0.018 (2)	0.016 (2)
C8	0.051 (3)	0.034 (3)	0.052 (3)	0.004 (2)	0.015 (2)	0.014 (2)
C7	0.056 (3)	0.031 (2)	0.039 (3)	0.006 (2)	0.017 (2)	0.010 (2)
C14	0.046 (3)	0.037 (2)	0.033 (2)	0.005 (2)	0.016 (2)	0.010 (2)
C15	0.046 (3)	0.059 (3)	0.040 (3)	0.004 (2)	0.014 (2)	0.017 (2)
C16	0.050 (3)	0.070 (4)	0.045 (3)	0.001 (3)	0.022 (2)	0.018 (3)
C18	0.049 (3)	0.048 (3)	0.045 (3)	0.004 (2)	0.018 (2)	0.021 (2)
C17	0.064 (3)	0.048 (3)	0.047 (3)	−0.002 (3)	0.029 (3)	0.018 (2)
N6	0.054 (3)	0.042 (2)	0.041 (2)	0.0064 (19)	0.0178 (19)	0.0175 (18)

### Geometric parameters (Å, °)

**Table:**

Cu1—I1	2.6550 (7)	C3—H3B	0.9300
Cu1—I1^i^	2.6579 (8)	C13—C12	1.394 (7)
Cu1—N1	2.037 (4)	C13—C14	1.477 (6)
Cu1—N6^ii^	2.060 (4)	C12—C11	1.379 (7)
Cu1—Cu1^i^	2.6935 (14)	C12—H12A	0.9300
S1—C9	1.779 (5)	C4—H4A	0.9300
S1—S2	2.0183 (17)	N4—C11	1.327 (6)
S2—C10	1.778 (5)	C11—H11A	0.9300
N2—C9	1.323 (5)	C2—H2B	0.9300
N2—C6	1.352 (6)	C8—C7	1.367 (6)
C5—C1	1.390 (6)	C8—H8A	0.9300
C5—C4	1.389 (6)	C7—H7A	0.9300
C5—C6	1.466 (6)	C14—C18	1.379 (7)
N3—C9	1.329 (6)	C14—C15	1.392 (7)
N3—C8	1.329 (6)	C15—C16	1.376 (7)
N1—C2	1.333 (6)	C15—H15A	0.9300
N1—C1	1.330 (5)	C16—C17	1.349 (7)
N5—C13	1.324 (6)	C16—H16A	0.9300
N5—C10	1.328 (6)	C18—N6	1.336 (6)
C1—H1A	0.9300	C18—H18A	0.9300
C6—C7	1.391 (6)	C17—N6	1.344 (7)
C10—N4	1.325 (6)	C17—H17A	0.9300
C3—C2	1.370 (7)	N6—Cu1^iii^	2.060 (4)
C3—C4	1.368 (6)		
			
Cu1—I1—Cu1^i^	60.92 (3)	C12—C13—C14	123.0 (4)
N1—Cu1—N6^ii^	115.68 (16)	C11—C12—C13	117.4 (4)
N1—Cu1—I1	106.36 (10)	C11—C12—H12A	121.3
N6^ii^—Cu1—I1	106.25 (12)	C13—C12—H12A	121.3
N1—Cu1—I1^i^	105.02 (11)	C3—C4—C5	119.8 (4)
N6^ii^—Cu1—I1^i^	104.97 (12)	C3—C4—H4A	120.1
I1—Cu1—I1^i^	119.08 (3)	C5—C4—H4A	120.1
N1—Cu1—Cu1^i^	122.23 (11)	C10—N4—C11	113.9 (4)
N6^ii^—Cu1—Cu1^i^	122.06 (12)	N4—C11—C12	123.3 (5)
I1—Cu1—Cu1^i^	59.59 (2)	N4—C11—H11A	118.3
I1^i^—Cu1—Cu1^i^	59.49 (3)	C12—C11—H11A	118.3
C9—S1—S2	104.18 (16)	N1—C2—C3	122.6 (4)
C10—S2—S1	104.60 (17)	N1—C2—H2B	118.7
C9—N2—C6	116.6 (4)	C3—C2—H2B	118.7
C1—C5—C4	116.6 (4)	C7—C8—N3	123.1 (4)
C1—C5—C6	119.6 (4)	C7—C8—H8A	118.5
C4—C5—C6	123.8 (4)	N3—C8—H8A	118.5
C9—N3—C8	114.2 (4)	C8—C7—C6	118.6 (4)
C2—N1—C1	117.8 (4)	C8—C7—H7A	120.7
C2—N1—Cu1	121.4 (3)	C6—C7—H7A	120.7
C1—N1—Cu1	120.8 (3)	C18—C14—C15	117.0 (4)
N2—C9—N3	128.4 (4)	C18—C14—C13	122.1 (4)
N2—C9—S1	110.9 (3)	C15—C14—C13	120.8 (4)
N3—C9—S1	120.7 (3)	C14—C15—C16	118.8 (5)
C13—N5—C10	116.9 (4)	C14—C15—H15A	120.6
N1—C1—C5	123.9 (4)	C16—C15—H15A	120.6
N1—C1—H1A	118.0	C17—C16—C15	120.0 (5)
C5—C1—H1A	118.0	C17—C16—H16A	120.0
N2—C6—C7	119.1 (4)	C15—C16—H16A	120.0
N2—C6—C5	116.7 (4)	N6—C18—C14	124.5 (5)
C7—C6—C5	124.2 (4)	N6—C18—H18A	117.8
N4—C10—N5	128.4 (4)	C14—C18—H18A	117.8
N4—C10—S2	120.6 (3)	C16—C17—N6	123.1 (5)
N5—C10—S2	111.1 (3)	C16—C17—H17A	118.5
C2—C3—C4	119.3 (4)	N6—C17—H17A	118.5
C2—C3—H3B	120.4	C18—N6—C17	116.6 (4)
C4—C3—H3B	120.4	C18—N6—Cu1^iii^	120.4 (4)
N5—C13—C12	120.0 (4)	C17—N6—Cu1^iii^	122.7 (3)
N5—C13—C14	117.0 (4)		
			
Cu1^i^—I1—Cu1—N1	118.18 (12)	C10—N5—C13—C12	0.7 (7)
Cu1^i^—I1—Cu1—N6^ii^	−118.04 (12)	C10—N5—C13—C14	−179.0 (4)
Cu1^i^—I1—Cu1—I1^i^	0.0	N5—C13—C12—C11	0.1 (7)
C9—S1—S2—C10	81.1 (2)	C14—C13—C12—C11	179.7 (4)
N6^ii^—Cu1—N1—C2	75.1 (4)	C2—C3—C4—C5	−0.6 (7)
I1—Cu1—N1—C2	−167.2 (3)	C1—C5—C4—C3	1.1 (6)
I1^i^—Cu1—N1—C2	−40.1 (4)	C6—C5—C4—C3	−178.3 (4)
Cu1^i^—Cu1—N1—C2	−103.2 (3)	N5—C10—N4—C11	0.0 (8)
N6^ii^—Cu1—N1—C1	−104.0 (4)	S2—C10—N4—C11	179.6 (4)
I1—Cu1—N1—C1	13.7 (4)	C10—N4—C11—C12	0.8 (8)
I1^i^—Cu1—N1—C1	140.8 (3)	C13—C12—C11—N4	−0.9 (8)
Cu1^i^—Cu1—N1—C1	77.7 (4)	C1—N1—C2—C3	1.0 (7)
C6—N2—C9—N3	−1.0 (7)	Cu1—N1—C2—C3	−178.1 (4)
C6—N2—C9—S1	178.0 (3)	C4—C3—C2—N1	−0.5 (7)
C8—N3—C9—N2	−0.2 (7)	C9—N3—C8—C7	1.0 (7)
C8—N3—C9—S1	−179.1 (3)	N3—C8—C7—C6	−0.6 (8)
S2—S1—C9—N2	176.0 (3)	N2—C6—C7—C8	−0.7 (7)
S2—S1—C9—N3	−4.8 (4)	C5—C6—C7—C8	179.6 (4)
C2—N1—C1—C5	−0.5 (7)	N5—C13—C14—C18	−173.4 (4)
Cu1—N1—C1—C5	178.7 (3)	C12—C13—C14—C18	6.9 (7)
C4—C5—C1—N1	−0.5 (7)	N5—C13—C14—C15	7.5 (7)
C6—C5—C1—N1	178.9 (4)	C12—C13—C14—C15	−172.2 (5)
C9—N2—C6—C7	1.4 (6)	C18—C14—C15—C16	−0.2 (7)
C9—N2—C6—C5	−178.8 (4)	C13—C14—C15—C16	178.9 (4)
C1—C5—C6—N2	−14.3 (6)	C14—C15—C16—C17	−0.6 (8)
C4—C5—C6—N2	165.0 (4)	C15—C14—C18—N6	0.0 (7)
C1—C5—C6—C7	165.5 (4)	C13—C14—C18—N6	−179.1 (4)
C4—C5—C6—C7	−15.2 (7)	C15—C16—C17—N6	1.6 (8)
C13—N5—C10—N4	−0.8 (7)	C14—C18—N6—C17	0.9 (7)
C13—N5—C10—S2	179.6 (3)	C14—C18—N6—Cu1^iii^	−174.0 (4)
S1—S2—C10—N4	−4.1 (4)	C16—C17—N6—C18	−1.7 (8)
S1—S2—C10—N5	175.5 (3)	C16—C17—N6—Cu1^iii^	173.0 (4)

Symmetry codes: (i) −*x*+1, −*y*+2, −*z*; (ii) *x*, *y*+1, *z*−1; (iii) *x*, *y*−1, *z*+1.
